# Baicalin and Geniposide Inhibit Polarization and Inflammatory Injury of OGD/R-Treated Microglia by Suppressing the 5-LOX/LTB4 Pathway

**DOI:** 10.1007/s11064-021-03305-1

**Published:** 2021-04-23

**Authors:** HuiMin Li, Yan Wang, Bin Wang, Min Li, JiPing Liu, HongLian Yang, YongHeng Shi

**Affiliations:** 1 Key Laboratory of Pharmacodynamic Mechanism and Material Basis of Traditional Chinese Medicine, Shaanxi Provincial Administration of Traditional Chinese Medicine, Shaanxi University of Chinese Medicine, Xianyang, 712046 China; 2 Affiliated Hospital of Shaanxi University of Chinese Medicine, Xianyang, 712046 China

**Keywords:** Cerebral ischemic, Baicalin, Geniposide, Leukotriene B4, Microglia, Inflammation

## Abstract

Cerebral ischemia causes severe neurological disorders and neuronal dysfunction. Baicalin (BC), geniposide (GP), and their combination (BC/GP) have been shown to inhibit post-ischemic inflammatory injury by inhibiting the 5-LOX/CysLTs pathway. The aims of this study were to observe the inhibitory effects of BC/GP on the activation of microglial cells induced by oxygen glucose deprivation and reoxygenation (OGD/R) and to investigate whether the 5-LOX/LTB4 pathway was involved in these effects. Molecular docking showed that BC and GP exhibited considerable binding activity with LTB4 synthase LTA4H. BV-2 microglia were transfected with a 5-LOX overexpression lentiviral vector, and then OGD/R was performed. The effects of different concentrations of BC, GP, and BC/GP (6.25 μM, 12.5 μM, and 25 μM) on cell viability and apoptosis of microglia were evaluated by MTT and flow cytometry. The expression of TNF-α, IL-1β, NF-κB, and pNF-κB also was measured by ELISA, Western blots and immunofluorescence. Western blots and qRT-PCR analysis were used to determine the levels of CD11b, CD206, and 5-LOX pathway proteins. Results showed that BC, GP, and BC/GP reduced the apoptosis caused by OGD/R in a dose-dependent manner, and cell viability was significantly increased at a concentration of 12.5 μM. OGD/R significantly increased the release of TNF-α, IL-1β, NF-κB, pNF-κB, and CD11b. These effects were suppressed by BC, GP, and BC/GP, and the OGD/R-induced transfer of NF-κB p65 from the ctytoplasm to the nucleus was inhibited in microglia. Interestingly, the LTB4 inhibitor, U75302, exhibited the same effect. Also, BC, GP, and BC/GP significantly reduced the expression of 5-LOX pathway proteins. These results demonstrated that BC/GP inhibited OGD/R-induced polarization in BV2 microglia by regulating the 5-LOX/LTB4 signaling pathways and attenuating the inflammatory response. Our results supported the theoretical basis for additional in-depth study of the function of BC/GP and the value of determining its unique target, which might provide a new therapeutic strategy for ischemic cerebrovascular disease.

## Introduction

Ischemic cerebral vascular disease (ICVD) involves acute occlusive lesions of blood vessels due to thromboembolism events that result in a decrease or interruption of the blood supply to specific regions of the brain. ICVD is a major cause of mortality and morbidity worldwide [1]. Ischemia can lead to a cascade of physiological and pathological reactions. The primary event is the activation of the inflammatory cascade that contributes to secondary processes, which ultimately result in brain injury [2–4]. Mounting evidence indicates that brain ischemia triggers inflammatory responses and leads to microglia activation, which produces a range of cytotoxic substances including TNF-α, IL-1β, iNOS, and other pro-inflammatory mediators that cause additional neuronal damage [5–7]. After ischemia, leukocytes infiltrate through the blood‐brain barrier (BBB) to the injured tissue and mediate the release of cytokines that further damage brain tissues [8]. Leukotriene B4 is produced from arachidonic acid (AA) through the sequential actions of 5-lipoxygenase (5-LOX), 5-lipoxygenase-activating protein (FLAP), and leukotriene A4 hydrolase (LTA4H) [9], and has a strong chemotactic effect on neutrophils and other leukocytes.

Huanglian jiedu decotion is a traditional Chinese medicine (TCM) compound composed of *Rhizoma coptidis* (huanglian), *Radix scutellariae* (huangqin), *Cortex phellodendri* (huangbo), and *Fructus Gardeniae* (zhizi). It is widely used for clearing heat dampness and to purge fire detoxification. Huanglian jiedu decotion has been used widely in the clinical treatment of a range of cardiovascular and cerebrovascular diseases. Huangqin and zhizi, which are traditional Asian herbs, also are used to clear heat dampness and purge fire detoxification. As the main active ingredient of huangqin, baicalin (BC), has been proven to have anti-bacterial and anti-inflammatory properties. It is widely used in the treatment of enteritis [10, 11], influenza [12–14], cerebrovascular diseases [15, 16]. BC has been shown to reduce cerebral infarct volume, and the pathological impairment of ischemic brain tissue has been alleviated in rats with cerebral ischemia, suggesting it has a neuroprotective effect on cerebral ischemia [17, 18]. Geniposide (GP), the main active ingredient of zhizi, elicits neuroprotective effects by alleviating inflammatory responses, and oxidative damage [19]. GP alleviates cognitive deficits by attenuating cholinergic defects and amyloidosis, as seen in a middle-aged Alzheimer model [20]. GP Geniposide treatment Perinatal hypoxic-ischemia mice significantly inhibited cell apoptosis, reduced serum IgG leakage into brain tissue, attenuated microgliosis, prevented loss of pericytes, loss of tight junction and adherens junction proteins[21]. GP in combination with ginsenoside Rg1 protected against focal cerebral ischemia in rats through inhibition of microglial microRNA following ischemic injury[22].

Our research team demonstrated that BC and GP in combination (BC/GP, 7:3) might ameliorate cerebral ischemic injury by inhibiting the 5-LOX/cysteinyl leukotrienes (CysLTs) pathway [23, 24]. It was proposed that inhibition of this pathway reduced inflammatory cell infiltration in ischemic areas through down-regulation of tumor necrosis factor-α (TNF-α) and interleukin-beta (IL-1β), as well as up-regulating transforming growth factor-beta (TGF-β) and interleukin-10 (IL-10) [25]. However, it is unclear whether the protective effect of BC/GP on cerebral ischemia occurs via 5-LOX/leukotriene B4 (LTB4).

In the present study, we hypothesized that the 5-LOX/LTB4 pathway could be a therapeutic target for BC/GP (7:3) in cerebral ischemia–reperfusion injury. Therefore, network pharmacology and molecular docking were used to verify the protective mechanism of BC, GP, and BC/GP on cerebral ischemic injury. We investigated whether the neuroprotective effects of BC/GP were accomplished by regulating the 5-LOX/LTB4 pathway and reduced production of pro-inflammatory factors in 5-LOX overexpressing microglia after oxygen glucose deprivation and reoxygenation (OGD/R).

## Materials and Methods

### Data Acquisition

All of the constituent data for huangqin and zhizi were obtained from the Traditional Chinese Medicine Systems Pharmacology Database and Analysis Platform (TCMSP, http://lsp.nwu.edu.cn/tcmsp.php) [26]. The bioactive ingredients of huangqin and zhizi were obtained from PubMed (http://www.ncbi.nlm.nih.gov/pubmed), CNKI (https://www.cnki.net/), and Springer (https://link.springer.com/) databases. The active components and targets were further identified using the parameters of the oral bioavailability (OB) threshold that was greater than or equal to 20% and a drug likeness (DL) that was greater than or equal to 0.18. FDA-approved targets for anti-cerebral ischemic injury drugs were collected from the Therapeutic Target Database (TTD) (http://bidd.nus.edu.sg/group/cjttd/), DrugBank (https://www.drugbank.ca/), Gene Cards (https://www.genecards.org/), and DisGeNET (http://www.disgenet.org/) databases. The targets obtained for huangqin and zhizi and the prediction targets for cerebral ischemia were mapped into Venny 2.1.0 (https://bioinfogp.cnb.csic.es/tools/venny/index.html) to obtain the anti-ischemic targets for huangqin and zhizi.

To elucidate the pathways targeted by the compounds and any gene-associated diseases, the identified genes were further analyzed using Integrated Discovery (DAVID, https://david.ncifcrf.gov/). A threshold count greater than or equal to 2 and EASE scores less than or equal to 0.05 were chosen for functional annotation clustering.

### Network Construction and Analysis

The network was built using Cytoscape 3.4.0 software and the network analyzer plugin. After analyzing the compound-target and target-pathway networks, it was concluded that huangqin and zhizi had anti-cerebral ischemic injury effects.

### Molecular Docking

The bioactive components of huangqin and zhizi were screened using Cytoscape analysis and Pubmed, CNKI, and Springer databases. The molecular structure was downloaded using TCMSP and PubChem (https://pubchem.ncbi.nlm.nih.gov/). The LTA4 hydrolase (LTA4H) protein structure was downloaded using the PDB (http://www.rcsb.org/pdb/ home/home.do) database. LTA4H was considered the synthase enzyme for LTB4. LTA4H was selected for docking since there was no protein conformation of LTB4 in the database. Protein preparation and subsequent molecular docking were performed using Discovery Studio 2.5 and Autodock Vina [27].

### Drugs and Reagents

The sources for drugs and reagents used in the study included Human HEK293T and BV2 microglia (CTCC BIOSCIENCE), baicalin and geniposide (Solarbio Bio Inc. Beijing, China, content ≥ 98%); dimethyl sulfoxide (DMSO, Sigma), MTT reagent (Sigma, USA), DMEM glucose medium (GIBCO BRL, USA), fetal bovine serum (FBS, GIBCO, USA), mouse TNF-α and IL-lβ ELISA Kits (Tianjin sel biotechnology co. Ltd, China), Earle's liquid (GIBCO), SYBR Premix Ex Taq Kits (TaKaRa, Japan), as well as montelukast and U75302 (Chengdu huana chemical preparation co. Ltd, China).

### Synthesis of the pHBLV-CMV-MCS-3flag-EF1-puro/Alox5 Plasmid

**Fig. 1:**
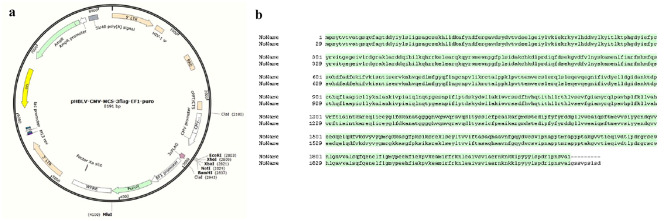
**a** pHBLV-CMV-MCS-3flag-EF1-puro/Alox5 plasmid; **b** comparison of Alox5 sequencing results

The map of the pHBLV-CMV-MCS-3flag-EF1-puro plasmid vector is shown in Fig. 1a. The Alox5 sequence was directly synthesized into the pHBLV-CMV-MCS-3flag-EF1-puro vector. Figure 1b demonstrates that the entire gene of the pHBLV-CMV-MCS-3flag-EF1-puro/Alox5 plasmid was synthesized successfully.

### BV2 Cell Virus Transfection

293 T cells were cultured in DMEM basal culture medium with 10% FBS and 1% penicillin–streptomycin in an incubator at 37 °C and 5% CO_2_. The 293 T cells were transfected with the phBL-CMV-MCS-3flag-EF1-PURo plasmid vector for lentivirus packaging. The supernatant was collected, and the virus particles were concentrated. BV2 microglia were incubated in 2 ml antibiotic-free DMEM overnight. Then 1 ml of the diluted viral mixture was added and the cells were incubated at 37 ℃ for 24 h. The medium was changed to FBS/DMEM 24 h later. The lowest cell lethal concentration was screened by adding the drug, G418. The drug concentration gradient was set at 200 μg/ml, 300 μg/ml, 400 μg/ml, and 500 μg/ml. Between the third day and the eighth day after adding G418, numerous cells began to die. The surviving cells formed monoclonal cultures, which were cultured further.

### Cell Culture and Treatment

BV2 microglia were transfected with the 5-LOX virus and cultured in medium with 10% FBS, 1% penicillin–streptomycin in an incubator at 37 °C and 5% CO_2_. When the BV2 cells had been passaged to the third generation, the culture medium was changed to a sugar-free Earle's solution. Cells were pretreated with various concentrations of BC, GP, BC/GP (7:3), U75302 (5 μM), and montelukast (5 μM) for 4 h. Subsequently, OGD/R (oxygen–glucose deprivation for 2 h and reperfusion for 12 h) was carried out at 37 °C using a three-gas incubator (94% nitrogen, 5% carbon dioxide, and 1% oxygen).

### Cell Viability Analysis

Cells were added to a 96-well plate at a concentration of 5000 cells/well. The MTT solution (5 mg/mL, Sigma) was added to the culture medium after stimulation. The cells were kept in a humidified incubator with 95% air and 5% CO_2_ at 37 ℃ for 4 h. The absorbance at 570 nm was measured using a microplate reader. The experimental groups are listed in Table 1.

**Table 1:** Experimental groups and drug doses

Groups	Drug administration
Control group	BV2 cells (virus-free), no OGD/R
OGD/R group Model group	BV2 cells (virus-free), OGD/R
	BV2 cells + 5-LOX virus, OGD/R
Drug groups	BV2 cells + 5-LOX virus + BC (6.25 μM), OGD/R
	BV2 cells + 5-LOX virus + BC (12.5 μM), OGD/R
	BV2 cells + 5-LOX virus + BC (25 μM), OGD/R
	BV2 cells + 5-LOX virus + GP (6.25 μM), OGD/R
	BV2 cells + 5-LOX virus + GP (12.5 μM), OGD/R
	BV2 cells + 5-LOX virus + GP (25 μM), OGD/R
	BV2 cells + 5-LOX virus + BC/GP (6.25 μM), OGD/R
	BV2 cells + 5-LOX virus + BC/GP (12.5 μM), OGD/R
	BV2 cells + 5-LOX virus + BC/GP (25 μM), OGD/R

### Apoptosis Assay

The cells were stained with propidium iodide (PI) and fluorescein isothiocyanate (FITC)-conjugated Annexin V stain (BD Bioscience, USA) to analyze cell apoptosis. The cells were washed three times using phosphate-buffered saline (PBS), then stained with PI in the dark at room temperature for 20 min. The cells were analyzed for cell cycle stages using a Canto II flow cytometer (BD Bioscience, USA). The cell groups that were assessed are shown in Table 1.

### Enzyme-Linked Immunosorbent Assay (ELISA)

After BV2 microglia were transfected with the 5-LOX virus, the cells were treated with OGD/R in 24-well plates. The supernatant was collected. The concentrations of inflammatory factors, TNF-α and IL-1β, were determined using ELISA kits according to the manufacturer’s instructions. The assays were standardized based on cell protein concentrations. The experimental groups and drug concentrations that were used are shown in Table 2.

**Table 2:** Experimental groups and drug concentrations

Groups	Drug administration
Control group	BV2 cells (virus-free), no OGD/R
OGD/R group Model groups Positive drug groups Drug groups	BV2 cells (virus-free), OGD/R
	BV2 cells + 5-LOX virus, OGD/R
	BV2 cells + 5-LOX virus + Montelukast, OGD/R BV2 cells + 5-LOX virus + U75302, OGD/R
	BV2 cells + 5-LOX virus + BC (12.5 μM), OGD/R
	BV2 cells + 5-LOX virus + GP (12.5 μM), OGD/R
	BV2 cells + 5-LOX virus + BC/GP (12.5 μM), OGD/R

### RT-PCR

RNA was extracted from BV2 cells using Trizol reagent (Invitrogen, Waltham, MA, USA). The total RNA was isolated using a reverse transcription kit according to the manufacturer’s instructions. The first-strand cDNA was prepared from the total RNA (1 μg) using reverse transcription (RT). The cDNA was amplified using SYBR Premix Ex Taq (Takara, Japan) with specific primers. The primer sequences for the specific genes are shown in Table 3.

**Table 3:** RT-PCR primer sequences

Gene	Forward (5′ → 3′)	Reverse (3′ → 5′)
Alox5-S	CTTTATTCTATTTATGCT	TTGGTGGGGGTGGGAGT
LTB4 CD206 CysLTs	AGGAGCCACTTCTCTGGTGA ATACCTTTGATGAATACA CTTGAACGTACTCTGACACTACAA	GCAGCTTCTGAAACCCAGTC GTCCTGAAAATACCCTGAGTGG GAGATGTCGTCAGATTTTCAGTTCCAT
CD11b	CATCCCATCTTTCCCGCTAATTCTG	TCGGTCCTGGACACGTTGTTCTCA
β-actin	CATGTACGTTGCTATCCAGG	CTCCTTAATGTCACGCACGAT

### Western Blots

Proteins were extracted using RIPA lysis buffer. Protein quantification was measured using a BCA Protein Assay Kit (Tianjin sel biotechnology co. Ltd) according to the manufacturer's instructions. The proteins (10 μg) were separated on a 10% SDS–polyacrylamide (PAGE) gel and transferred to a polyvinylidene difluoride (PVDF) membrane. The membrane was blocked with 5% skim milk for 1 h, washed three times (5 min each) with Tris-buffered saline plus Tween-20 (TBST). Each membrane was incubated with its respective primary antibody at 4 °C overnight, washed in TBST three times (8 min each) and incubated with secondary antibody for 2 h at room temperature, then washed again in TBST three times (8 min each). The protein bands were detected using electrochemiluminescence (ECL), and the band density was normalized using β-actin.

### Immunofluorescence Analysis

To determine the expression and localization of NF-κB p65 in the BV2 cells, double-labeled immunocytochemistry was performed according to a previously described protocol [7, 28].

### Statistical Analyses

All statistical analyses were performed using Graphpad Prism 5 software. Data were expressed as means ± SD from at least three independent experiments. The comparison of the same time points was performed by independent sample t-tests or analysis of variance. One-way analysis of variance was used and the data between the two groups were compared using the LSD test. P < 0.05 and P < 0.01 were considered statistically significant.

## Results

### Network Pharmacology Identified that Huangqin and Zhizi Might have Anti-inflammatory Effects on Cerebral Ischemia

**Fig. 2:**
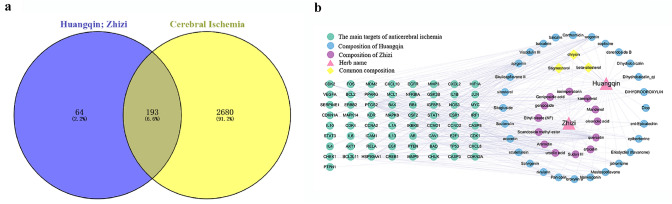
Network maps. **a** Mapping of huanglian, zhizi and cerebral ischemia targets. **b** Drug-target interaction network

Sixty-two chemical constituents were identified for huangqin, and 32 chemical constituents were identified for zhizi. Two hundred fifty-seven predicted targets were screened from TCMSP, TCMID, and other databases. The targets obtained for huangqin and, zhizi and the prediction targets for cerebral ischemia were mapped into Venny to obtain 193 anti-ischemic targets for huangqin and zhizi (Fig. 2a). The drug-target interaction networks (Fig. 2b) were visualized using Cytoscape. Thus, it was determined that baicalin (BC) and geniposide (GP) were the main components involved in anti-cerebral ischemia.

**Fig. 3:**
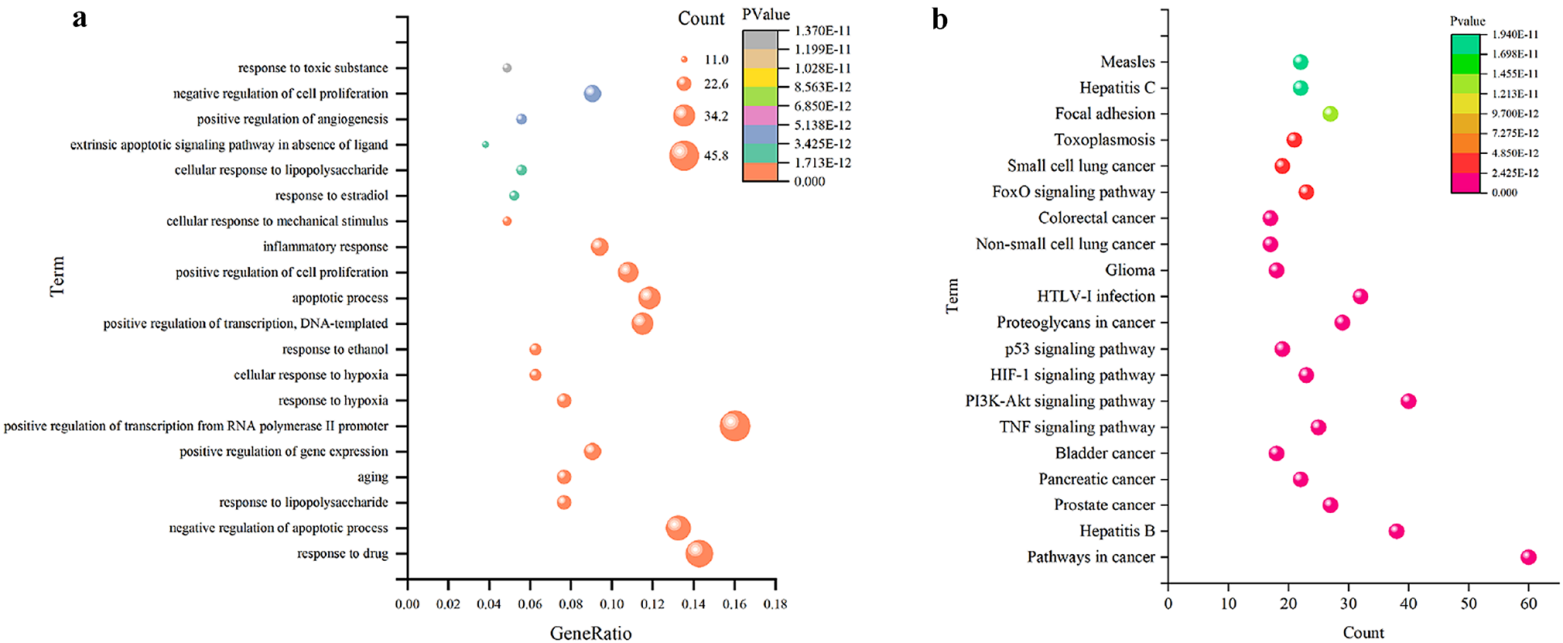
Enrichment analysis. **a** GO enrichment analysis; **b** KEGG enrichment analysis

The 193 anti-ischemic targets were associated with the following five functions, response to hypoxia, apoptosis, cell proliferation, inflammatory responses, and angiogenesis (Fig. 3a). KEGG (Fig. 3b) analysis revealed that these target proteins were involved in multiple signaling pathways. The identified pathways could be divided into four broad categories, including (1) cell proliferation and differentiation, (2) regulation of adhesion, aggregation, and migration of immune cells, (3) inflammatory factors and inflammatory mediators that directly mediated the regulation of cerebral ischemia inflammation, (4) regulation of receptors that mediated the expression of downstream inflammatory factors.

Through the analysis of GO-BP and KEGG, we determined that the anti-ischemic injury targets of huangqin and zhizi primarily acted on the inflammatory response. 5-LOX, CysLTs and LTB4, which are metabolites of arachidonic acid, were the common inflammatory targets of BC and GP. Based on our research team’s previous research, we carried out a follow-up study on the effects of BC and GP on LTB4.

### BC and GP Showed Good Binding Activity to LTA4H, a LTB4 Synthase

**Fig. 4:**
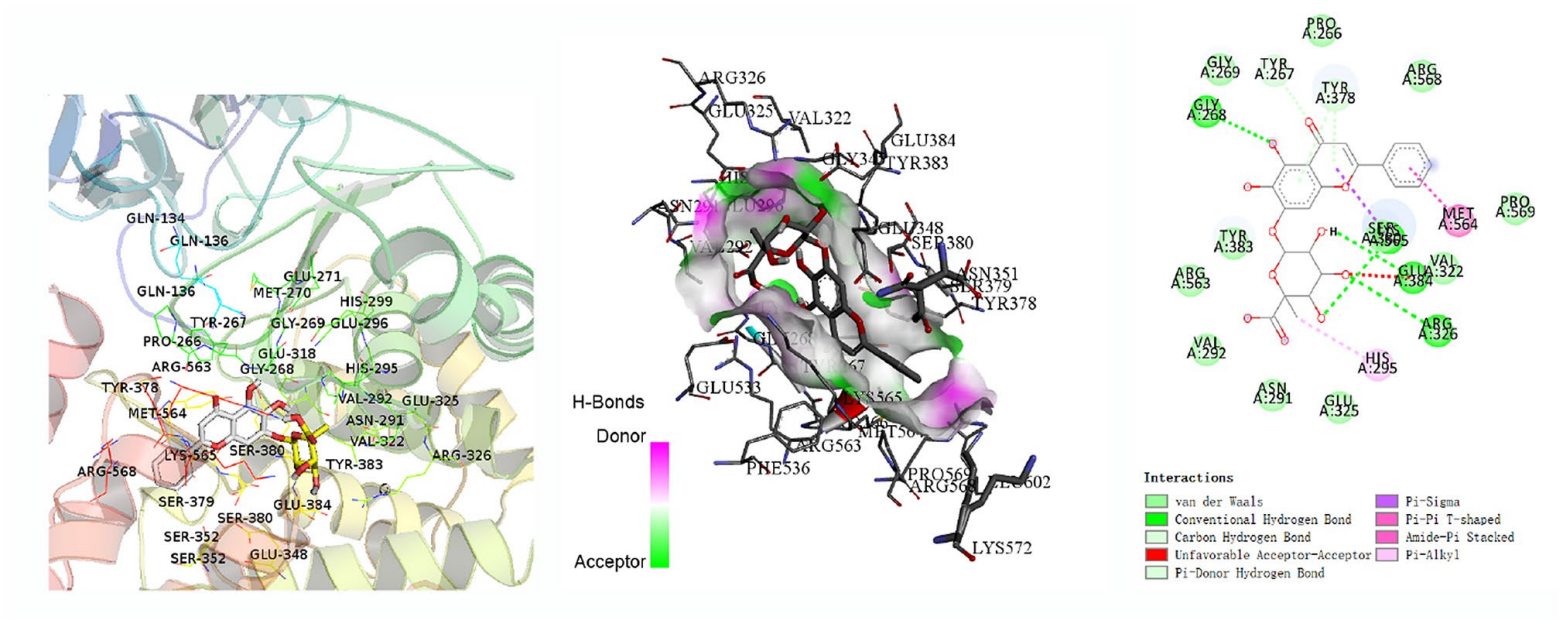
Schematic diagram of the optimal conformation and interaction between baicalin and LTA4H

**Fig. 5:**
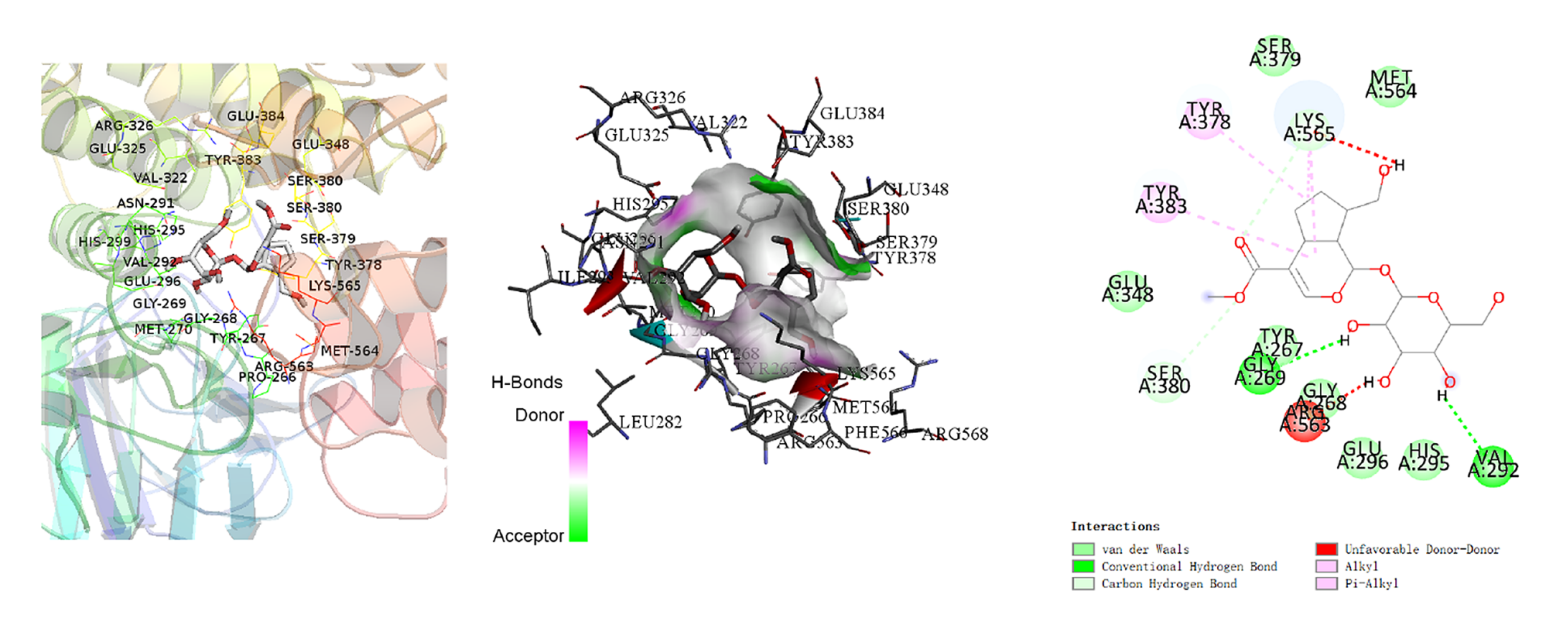
Schematic diagram of the optimal conformation and interaction between geniposide and LTA4H

The network topology was analyzed using the network analysis plugin. The components that were greater than the average value of Degree and BC were selected as the primary components of huangqin and zhizi that were active against cerebral ischemia. When the effects of the components in huangqin and zhizi on cerebral ischemia, the active components with higher concentrations in huangqin and zhizi were selected as docking molecules using the CNKI, PubMed, Springer databases. The antagonist for leukotriene B4, U75302, was selected as a reference. The parameter settings and docking results are shown in Table 4. Prediction of the binding activity with LTA4H for the compounds was based on the binding energy in kcal/mo. Binding energy that was greater than 7.0 indicated strong binding activity [29]. Molecular docking showed that the active ingredients in huangqin and zhizi had strong binding efficiency with LTA4H, and the conformations for baicalin and geniposide were plotted (Figs. 4, 5). We predicted that baicalin and geniposide (BC/GP) could inhibit LTB4.

**Table 4:** Docking parameters

Molecular	CAS	Protein	Affinity (kcal/mol)
U75302	119477-85-9	LTA4H (4MKT)	− 8.1
Baicalein	491-67-8		− 8.9
Baicalin	21967-41-9		− 9.2
Geniposide	24512-63-8		− 7.5

### BC/GP Reduced Apoptosis, and 12.5 μM BC/GP Improved Cell Viability in BV2 Microglia Cells Exposed to OGD/R

**Fig. 6:**
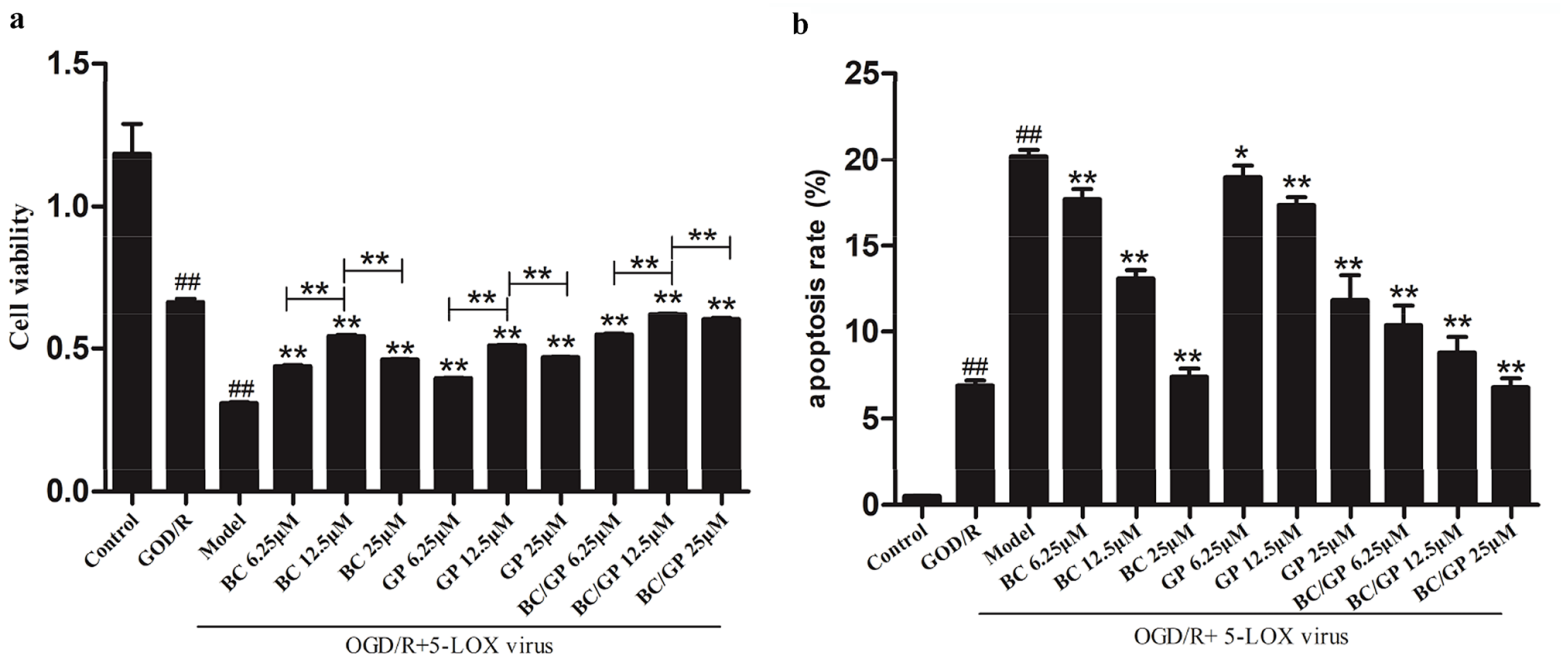
Effects of different concentrations of BC, GP and BC/GP (7:3) on cell viability and apoptosis. **a** MTT was used to detect cell activity. **b** Apoptosis was detected using flow cytometry. Each value indicates the mean ± SD (n = 6). ^##^*p* < 0.01 compared to the control group, **p < 0.01 compared to the model group

After BV2 microglia were transfected with the 5-LOX virus and exposed to the OGD/R environment, we observed that the rate of apoptosis in microglia was significantly higher than the control and OGD/R only groups (*p* < 0.01, Fig. 6a, b). After treatment with different concentrations of BC, GP, and BC/GP (7:3), the rate of apoptosis was significantly reduced in a dose-dependent manner (*p* < 0.01, Fig. 6b). When the microglia that overexpressed 5-LOX were exposed to OGD/R, the microglia exhibited significantly lower cell viability than the control group (*p* < 0.01, Fig. 6a). At concentrations of 6.25 μM, 12.5 μM, and 25 μM, BC, GP, and BC/GP (7:3) significantly increased cell viability compared with the model (*p* < 0.01, Fig. 6a). The data revealed that the cell viability was optimal when the concentrations of BC, GP, and BC/GP (7:3) were 12.5 μM. The results also revealed a significant difference between with 6.25 μM and 25 μM concentrations (*p* < 0.01, Fig. 6a). Thus, the concentration of 12.5 μM was used in later experiments.

### BC/GP Relieved OGD/R-Induced Inflammatory Damage in BV2 Microglia

**Fig. 7:**
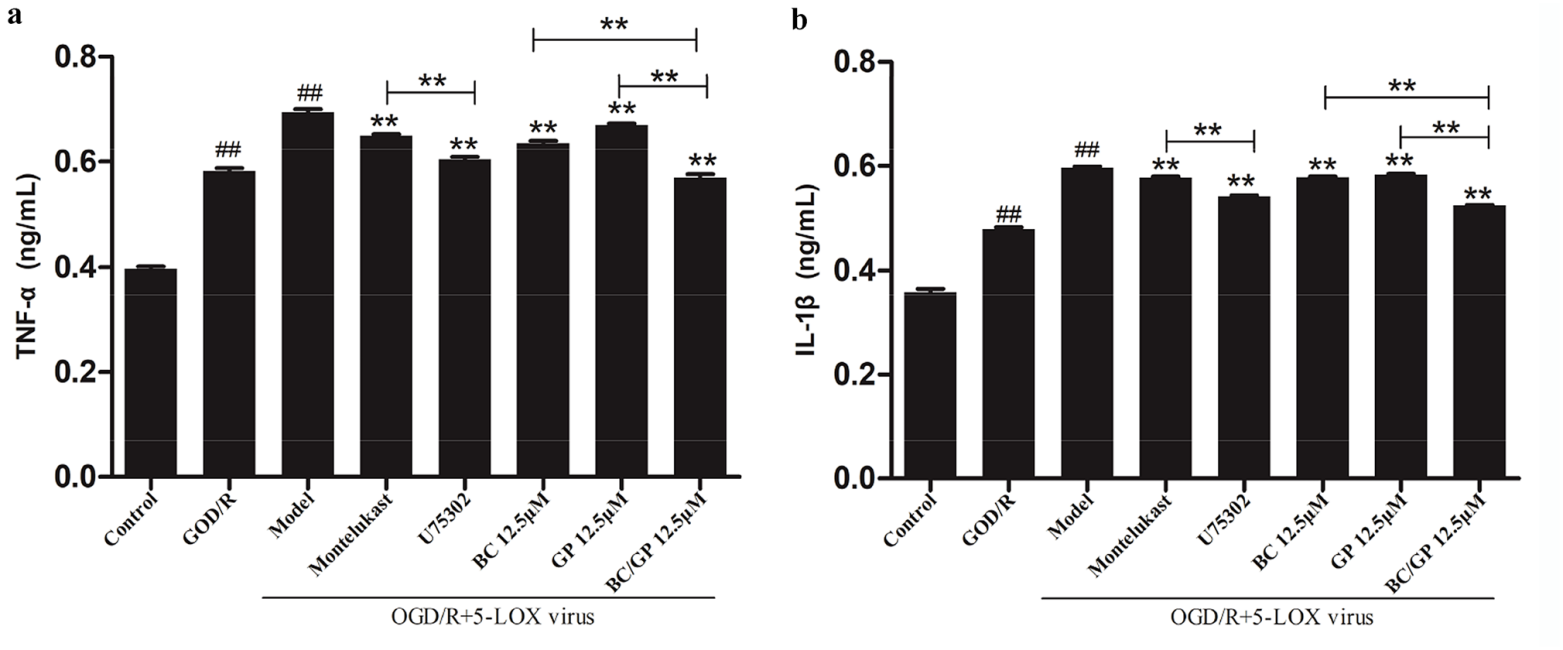
BC/GP relieved OGD/R induced inflammatory damage. U75302 is an antagonist of LTB4. Montelukast is an antagonist of CysLTs. **a** The concentration of TNF-α was tested using an ELISA. **b** The concentration of IL-1β was tested using an ELISA. Each value indicates the mean ± SD (n = 3). ^##^p < 0.01 compared to the control group, **p < 0.01 compared to the model group

When the microglia overexpressing 5-LOX were exposed to OGD/R, the levels of TNF-α (Fig. 7a) and IL-1β (Fig. 7b) released from the cells were significantly higher than in the control group (*p* < 0.01). However, BC, GP, and BC/GP (7:3) inhibited this increase (*p* < 0.01, Fig. 7). The combination of BC/GP had the greatest efficacy, and there was a significant difference when the effects of BC/GP were compared with the BC and GP groups (*p* < 0.01, Fig. 7). The effect of the LTB4 antagonist, U75032, was stronger than montelukast, a CysLTs antagonist (*p* < 0.01, Fig. 7). These data indicated that BC/GP and U75302 could alleviate OGD/R-induced inflammatory damage in BV2 microglia.

**Fig. 8:**
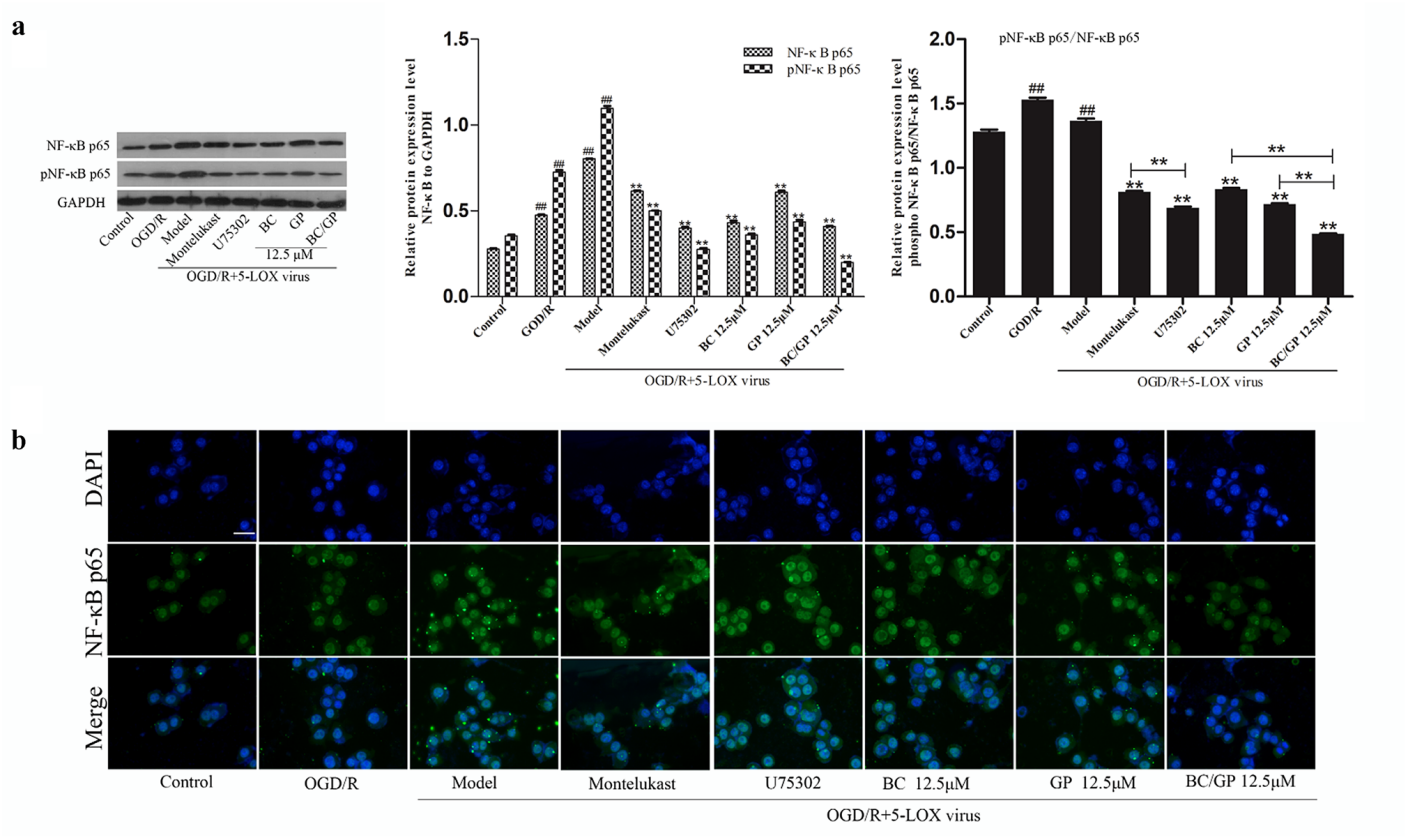
BC/GP inhibited NF-κB activation. U75302 is an antagonist of LTB4. Montelukast is an antagonist of CysLTs. **a** Protein levels of NF-κB p65 and pNF-κB p65 were analyzed using Western blots. **b** The cellular location of NF-κB p65 in 5-LOX overexpressing microglia exposed to OGD/R. Scale bar = 20 μm. Each value indicates the mean ± SD (n = 3). ^##^p < 0.01 compared to the control group, **p < 0.01 compared to the model group

Activation of NF-κB is a central event leading to inflammation, which is characterized by the translocation of p65 from the cytoplasm to the nucleus and the expression of phosphorylated p65. The Western blot analysis indicated that when microglia overexpressing 5-LOX were exposed to OGD/R, the expression level of phosphorylated NF-κB p65 was significantly higher than the control and OGD/R groups (*p* < 0.01, Fig. 8a). NF-κB p65 phosphorylation levels were significantly reduced after treatment with BC, GP, and BC/GP (7:3). pNF-κB p65/ NF-κB p65 levels were significantly lower than that of the BC and GP groups after BC/GP treatment (*p* < 0.01, Fig. 8a), indicating the efficacy of BC/GP was superior to that of BC and GP. The phosphorylation level of NF-κB p65 after U75302 treatment was significantly lower than that of the montelukast group (*p* < 0.01, Fig. 8a).

We also examined the localization of NF-κB p65 in BV2 cells in different conditions using double-labeled immunofluoresce. As shown in Fig. 8b, NF-κB p65 was expressed primarily in the cytoplasm (green) in the control group. However, it was translocated to the nucleus in the model group (Fig. 8). Merged images (blue/green) indicated that OGD/R induced much of the NF-κB p65 protein transfer from the cytoplasm to the nucleus. Treatment with 12.5 μM BC, GP, and BC/GP (7:3) markedly reversed NF-κB p65 immunostaining to nearly basal levels and redistributed it into the nucleus and cytoplasm. BC/GP (7:3) at the concentration of 12.5 μM exerted a much more robust inhibitory effect.

### BC/GP Inhibited the Expression of M1 and M2 Markers in Microglia

**Fig. 9:**
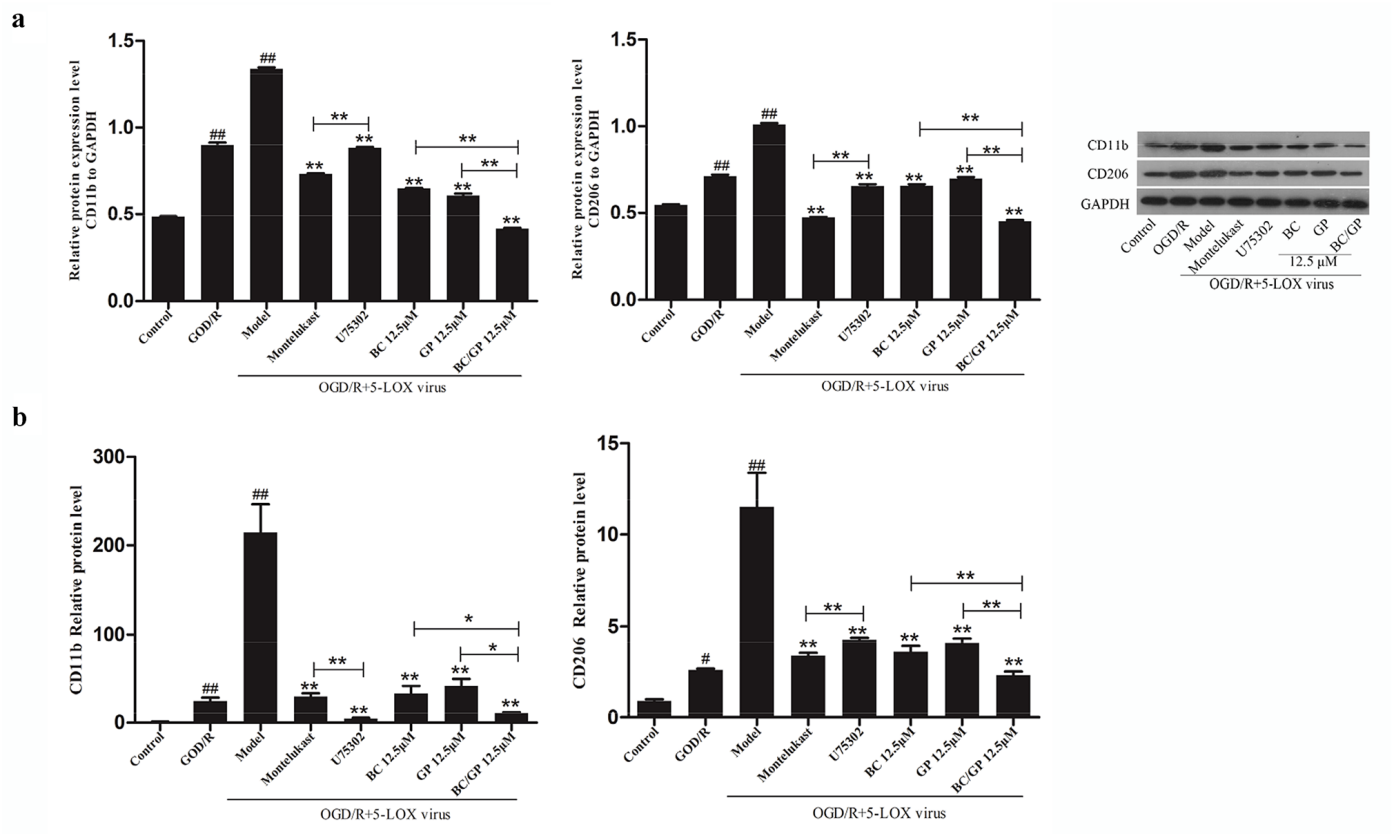
BC/GP inhibited microglial polarization. CD11b is a microglial M1 marker. CD206 is a microglial M2 marker. U75302 is an antagonist of LTB4. Montelukast is an antagonist of CysLTs. **a** Protein levels of CD11b and CD206 were analyzed using Western blots. **b** Expression of CD11b and CD206 was assessed using through qRT-PCR. Each value indicates the mean ± SD (n = 3). ^##^p < 0.01 compared to the control group, **p < 0.01 compared to the model group

To evaluate whether the neuroprotective effect of BC/GP (7:3) was associated with BV2 microglia polarization, we measured the expression of CD11b and CD206 using qRT-PCR and Western blots to determine the effect of BC/GP (7:3) on phenotype switches of the microglia. The qRT-PCR and Western blot analysis (Fig. 9a, b) revealed that when microglia overexpressing 5-LOX were exposed to OGD/R, the expression of the M1 marker, CD11b, was significantly higher than the control group (*p* < 0.01). After treatment with BC, GP, and BC/GP, the expression of CD11b was significantly lower than the model group. Compared with the BC and GP groups, the combination of BC/GP had the greatest effect and produced synergistic effects (*p* < 0.01). Compared with montelukast, the CD11b mRNA levels were significantly reduced after U75302 treatment (*p* < 0.01, Fig. 9b), but protein levels were increased based on the Western blot results (*p* < 0.01, Fig. 9a). We also observed that the expression of the microglia M2 marker, CD206, exhibited the same trend as CD11b (Fig. 9a, b). When microglia overexpressing 5-LOX were exposed to OGD/R, the expression of CD206 was significantly higher than the control and OGD/R groups (*p* < 0.01, Fig. 9a, b). After treatment with BC, GP and BC/GP, CD11b expression was significantly lower than the model group. The effect of BC/GP were more robust than BC and GP (p < 0.01). U75302 and Montelukast significantly reduced the expression of CD206 compared with the model group (p < 0.01). These results demonstrated that LTB4 regulated microglia polarization.

### BC/GP Exhibited an Inhibitory Effect on the 5-LOX Pathway

**Fig. 10:**
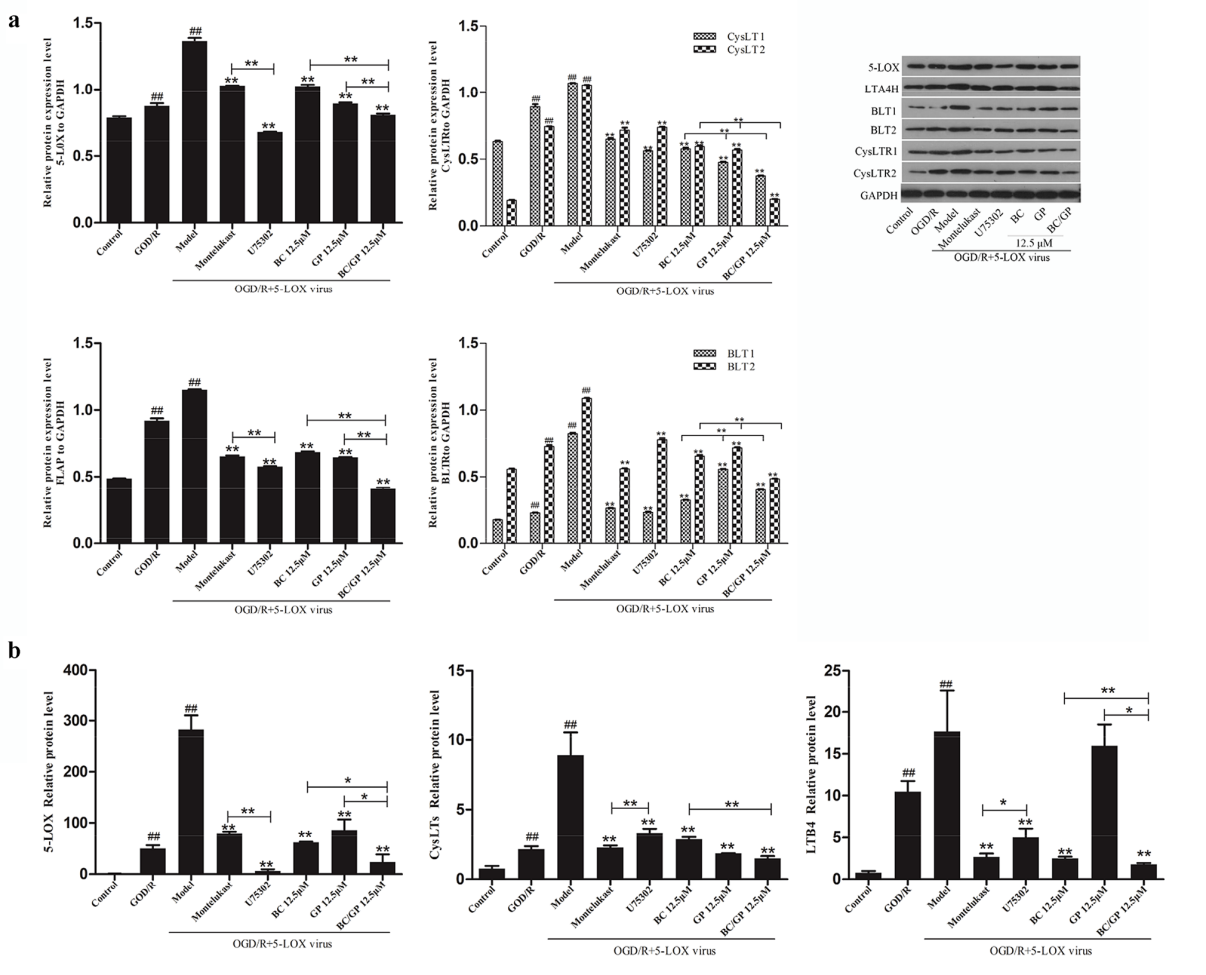
Effects of BC/GP on the 5-LOX pathway. U75302 is an antagonist of LTB4. Montelukast is an antagonist of CysLTs. CysLT1 and CysLT2 are two receptors for CysLTs. BLT1 and BLT2 are two receptors for LTB4. LTA4H is a synthetase of LTB4. **a** Expression of the 5-LOX pathway was accessed using Western blots. **b** The related protein expression of the 5-LOX pathway was analyzed using qRT-PCR. Each value indicates the mean ± SD (n = 3). ^##^p < 0.01 compared to the control group, *p < 0.05 compared to the model group, **p < 0.01 compared to model group

To further clarify the role of BC/GP, we assessed the expression of 5-LOX pathway proteins using Western blots and qRT-PCR. The Western blots and qRT-PCR results showed that when microglia overexpressing 5-LOX were exposed to OGD/R, the expression of 5-LOX was significantly increased compared with the control group (*p* < 0.01, Fig. 10a, b). After treatment with BC, GP, and BC/GP, the expression of 5-LOX was significantly lower than the model group. Compared with BC and GP groups, the combination of BC/GP (7:3) had the greatest effect and produced synergistic effects (*p* < 0.01, Fig. 10a, b). Compared with montelukast group, the U75302 group displayed significantly reduced expression of 5-LOX (*p* < 0.01, Fig. 10a, b). This suggests that the LTB4 pathway might play a dominant role in the elevated expression of 5-LOX.

Based on the qRT-PCR results, we determined that the expression of CysLTs and LTB4 mRNAs was significantly higher in the model group than the control and OGD/R groups (*p* < 0.01, Fig. 10b). Western blot results showed that the expression of CysLT1, CysLT2, BLT1, BLT2, and the LTB4 synthetase, LTA4H, were significantly higher in the model group than the control and OGD/R groups (*p* < 0.01, Fig. 10a). After treatment with BC, GP, and BC/GP, the expression of CysLTs mRNA, LTB4 mRNA, CysLT1, CysLT2, BLT1, BLT2, and LTA4H were significantly lower than the model group (*p* < 0.01, Fig. 10a, b). Compared with the BC and GP groups, the combination of BC/GP (7:3) had the greatest effect and produced synergistic effects (p < 0.01, Fig. 10a, b). This result indicated that BC/GP (7:3) might protect microglia from OGD/R damage by down-regulating the 5-LOX pathway. The effect of the combination of BC and GP was superior to BC and GP alone, and it exhibited a synergistic effect.

## Discussion

During cerebral ischemia, numerous inflammatory factors are produced that induce secondary injury and lead to neuronal damage. It has been reported that baicalin (BC) and geniposide (GP) exert protective effect on PC12 cells injured by OGD [30, 31]. Studies also have shown that baicalin prevented OGD-induced apoptosis of SH-SY5Y cells in culture and hippocampal cells, resulting in improved learning and memory impairment induced by global cerebral ischemia/reperfusion in gerbils [32]. Our reaserch team found earlier that BC and GP in combination (BC/GP) protected neurological function from injury, improved BBB permeability, and alleviated brain edema injury in a rat model of ischemia–reperfusion [33–35]. Our team also found that BC, GP, and BC/GP reduce inflammatory injury and tissue edema in the ischemic area of rats with cerebral ischemia [25, 36]. The underlying mechanism is related to the inhibition of microglia activation and the 5-LOX/CysLTs signal pathway [23]. Based on the search of the CNKI, PubMed, and Springer databases, we discovered that BC and GP were the active components of huangqin and zhizi. For the first time, we conducted a network pharmacologic analysis of huangqin and zhizi to determine the mechanism of action of BC and GP involved in the treatment of cerebral ischemia. We predicted that huangqin and zhizi protected against cerebral ischemic injury primarily through inhibiting inflammation. The results of the network pharmacology prediction were consistent with our previous study.

Increasing evidence has confirmed the ubiquitous role of neuroinflammation in the pathogenesis of neurological disorders, including Alzheimer’s disease (AD), Parkinson’s disease (PD), and ischemic stroke [37–39]. Microglia are intrinsic components of the brain immune system [40] and are activated in response to brain injury [41]. It is well known that activated microglia become hypertrophic, undergo rapid proliferation, and migrate to inflammatory sites where they produce excessive amounts of neurotoxic and pro-inflammatory mediators that produce neuronal damage [42–44]. On the other hand, apart from microglia, other glia cells, including astrocytes, also modulate the inflammatory response by releasing a number of pro/anti-inflammatory mediators and anti-oxidant molecules [45]. Depending on the milieu in which they become activated or the factors by which they are stimulated, microglia possess different states of activation, including classical activation, alternative activation, and acquired deactivation [46, 47]. Classic activation refers to M1 microglia, which are associated with the production of pro-inflammatory cytokines, including TNF-α, IL-6, nitric oxide (NO), and reactive oxygen species (ROS) [48]. Alternative activation is limited to the activation state induced by IL-4 or IL-13 and is closely associated with M2 genes that promote anti-inflammation and tissue repair [49]. Acquired deactivation is another state involved in alleviating acute inflammation. It is induced primarily by the uptake of apoptotic cells or exposure to anti-inflammatory cytokines, including IL-10 and transforming growth factor-β (TGF-β) [46, 47]. The general functional outcomes observed in acquired deactivation include inhibition of pro-inflammatory cytokine production, modification of inflammatory signaling pathways and increased expression of scavenger receptors thereby promoting debris clearance[47]. Following stimulation, microglia dynamically switch between M1 and M2 phenotypes resulting in neurotoxic and neuroprotective effects, respectively. Accordingly, a timely shift of microglial M1 to M2 phenotype has been regarded as a promising strategy for the management of neuroinflammation-related disorders [50]. Our research team established a model of BV2 microglia-activated inflammatory injury using in vitro LPS stimulation. We found that BC/GP inhibited the expression of NO, TNF-α, and IL-6. BC/GP also increased the expression of IL-10 and TGF-β to protect against neuronal injury after cerebral ischemia [51, 52]. Meanwhile, we discovered that BC/GP could inhibit the excessive activation of microglia through anti-inflammatory and neuroprotective effects and has protective effects on nerve injury following cerebral ischemia. Finally, BC/GP might be associated with inhibition of the expression of 5-LOX/CysLTs pathway [24]. In this study, we found that BC/GP inhibited both M1 polarization and M2 polarization of microglia. We stimulated BV2 cells three ways in the administration groups. ① After BV2 microglia were transfected with the 5-LOX virus overexpression of 5-LOX led to microglia activation. ② BC, GP, BC/GP (7:3) drug intervention caused microglia M1 to M2 phenotype. ③ Secondary damage of microglia caused by exposure to OGD/R environment. In the model group, expression of the microglia M2 marker, CD206, was significantly increased without drug intervention. Therefore, we speculated that microglia were in the process of dynamic transformation from the M1 phenotype to the M2 phenotype. However, additional verification is needed.

Leukotrienes (LTs) are short-lived, potent pro-inflammatory lipid mediators expressed in macrophages, neutrophils, and mast cells [53]. Arachidonic acid stimulated the production of two groups of leukotrienes under the action of 5-LOX. LTB4 was produced due to the action of LTA4 hydrolase, and CysLTs were produced under the action of LTC4 synthase. Each group of leukotrienes acted through their specific receptors (BLTR and CysLTR, respectively) [54]. CysLTs play an essential role in the pathogenesis of a range of CNS diseases such as cerebral ischemia and brain trauma [55]. CysLTs are potent inflammatory mediators in ischemic stroke. The cysteinyl leukotriene receptor 2 (CysLTR2) has been shown in vitro to mediate microglial activation and indirectly induces ischemic neuronal injury. It has been reported that inhibiting the expression of the 5-LOX/CysLTs pathway could reduce the toxicity of neuroglial activation and exert anti-inflammatory effects in cerebrovascular disease [56–58].

LTB4 has been reported to activate and recruit leukocytes [59] and T cells [60], which results in stimulating the production of cytokines and nuclear factor-kappaB (NF-κB) [61]. Excessive production of LTB4 results in robust production of chemokines and cytokines along with overwhelming neutrophil migration to the site of infection, where they release inflammatory mediators that cause tissue damage [62]. Thus, LTB4 is capable of maintaining the expression of inflammatory programs in monocytes and macrophages as well as prolonging neutrophil recruitment to the inflammatory foci [62]. Furthermore, when LTB4 binds to its receptor BLT1, it appears to impair the clearance of dead cells [63]. When apoptotic cells are not appropriately cleared, they become necrotic and secrete numerous toxic molecular components [64]. Inhibition of leukotriene B4 suppressed leukocyte infiltration after injury, thus reducing the expression of inflammatory factors TNF- α, IL-6, and IL-1β [65]. LTB4 phosphorylates MAPKs and stimulates NF-κB-dependent inflammation via BLTR in cultured monocytes [61]. It has been shown that the plasma and brain levels of LTB4 increased simultaneously after cerebral ischemia, and elevation of LTB4 levels increased the volumes of the cerebral infarctions [53]. Under the ischemic conditions following a stroke, tissue glutathione (GSH) is rapidly depleted [66]. GSH is a critical component in the synthesis of CysLTs, and GSH deficiency inhibits their synthesis of CysLTs. Therefore, at the levels that could occur in the ischemic brain, the LTB4-mediated effects might become more pronounced.

In this study, we verified that BC and GP exhibited reasonable binding activity on LTA4H of LTB4 synthase through molecular docking. We established the OGD/R model for microglia along with overexpression of 5-LOX. In a series of experiments, we found that compared with the montelukast group, U75302 significantly reduced the expression of inflammatory factors TNF-α, IL-1β and NF-κB by OGD/R-treated microglia. Western blots and qRT-PCR results revealed that U75302 significantly inhibited the expression of 5-LOX. Therefore, we speculated that when 5-LOX was highly expressed, the 5-LOX/LTB4 pathway might mediate a more severe inflammatory response. At the same time, U75302 reduced the expression of CD11b, a marker of M1-type microglia. However, the expression of CD206, a marker of M2-type microglia, was inhibited after drug administration. We speculated that when the microglia cells overexpressing 5-LOX were pretreated with U75302, montelukast, BC, GP, and BC/GP for 4 h and then exposed to the OGD/R environment, the microglia cells were in a state of acquired deactivation and then changed via the process of dynamic transformation into the M1/M2 states. The results showed that BC/GP significantly inhibited the expression of 5-LOX pathway proteins and inflammatory factors and inhibited the M1 polarization of microglia, which also had a synergistic effect.

In summary, BC/GP might alleviate OGD/R induced inflammatory damage of microglia through the 5-LOX/LTB4 pathway. Therefore, the 5-LOX/LTB4 pathway blockade is expected to dampen inflammatory responses and restore tissue homeostasis during inflammation, which might provide a new therapeutic strategy for ischemic cerebrovascular disease.

## Data Availability

All data used to support the findings of this study are included within the article.
